# Renal dysfunction contributes to deteriorated survival outcomes in patients with upper and lower gastrointestinal bleeding: insights from a cohort study of 1160 cases

**DOI:** 10.1038/s41598-025-87969-7

**Published:** 2025-01-30

**Authors:** Orsolya Anna Simon, Levente Frim, Nelli Farkas, Zoltán Sipos, Nóra Vörhendi, Eszter Boros, Dániel Pálinkás, Brigitta Teutsch, Patrícia Kalló, Vivien Vass, Andrea Szentesi, Roland Hágendorn, Péter Hegyi, Bálint Erőss, Imre Szabó

**Affiliations:** 1 First Department of Medicine, Medical School, University of Pécs, Ifjúság Útja 13, 7624 Pécs, Hungary; 2 Institute for Translational Medicine, Medical School, University of Pécs, Pécs, Hungary; 3 Institute of Bioanalysis, Medical School, University of Pécs, Pécs, Hungary; 4 Centre for Translational Medicine, Semmelweis University, Budapest, Hungary; 5 Institute of Pancreatic Diseases, Semmelweis University, Budapest, Hungary; 6 Internal Medicine, Hospital and Clinics of Siófok, Siófok, Hungary; 7 First Department of Internal Medicine, St. George University Teaching Hospital of County Fejér, Székesfehérvár, Hungary; 8 Department of Gastroenterology, Central Hospital of Northern Pest - Military Hospital, Budapest, Hungary; 9 Department of Radiology, Medical Imaging Centre, Semmelweis University, Budapest, Hungary; 10 Department of Primary Health Care, Department of Family Medicine, Medical School, University of Pécs, Pécs, Hungary

**Keywords:** Kidney function, Gastrointestinal bleeding, Survival, Mortality, Transfusion, Gastroenterology, Nephrology

## Abstract

Both acute kidney injury and chronic kidney disease are risk factors for many outcomes of gastrointestinal bleeding (GIB). These are associated with higher mortality, longer hospitalisation, and greater need for transfusion in case of overt GIB. Our study aimed to further evaluate the role of kidney function in several clinical outcomes of GIB patients. The Hungarian Gastrointestinal Bleeding Registry collected data on all-cause GIB between 2019 and 2022. A multi-level data-validation system provided high-quality data, which was retrospectively analysed. Numerous primary (in-hospital mortality, discharge, need for endoscopic intervention, in-hospital rebleeding, length of hospitalisation, need for emergency surgery, need for endoscopic examination and need for intensive care unit) and secondary (detection of *Helicobacter pylori* (*H. pylori*), recognition of cancer as the source of bleeding, need for any kind of transfusion or clotting factor, anticoagulation therapy) outcomes were observed. Descriptive statistical tools were used to summarize our data. Among others, estimated glomerular filtration rate (eGFR) (ml/min/1.73 m^2^) was used as continuous variable, mean, standard deviation, median, interquartile range and minimum/maximum values were calculated. Reduced kidney function was associated with in-hospital mortality (eGFR: 42.63 ± 28.78 ml/min/1.73 m^2^ vs. 57.08 ± 26.62 ml/min/1.73 m^2^, p < 0.001), need for red blood cells (RBC) transfusion (eGFR: 51.98 ± 27.90 ml/min/1.73 m^2^ vs. 60.11 ± 25.06 ml/min/1.73 m^2^, p < 0.001) and clotting factor supplementation (eGFR: 47.40 ± 27.41 ml/min/1.73 m^2^ vs. 56.68 ± 27.02 ml/min/1.73 m^2^, p < 0.001). Better eGFR values at admission were associated with discharge home after proper treatment, compared to any other outcome of the admission (eGFR: 58.12 ± 25.56 ml/min/1.73 m^2^ vs. 50.23 ± 29.69 ml/min/1.73 m^2^, p < 0.001), *H. pylori* positivity (eGFR: 59.63 ± 25.24 ml/min/1.73 m^2^ vs. 52.76 ± 25.44 ml/min/1.73 m^2^, p = 0.021) and the need for endoscopic intervention (eGFR: 58.65 ± 26.61 ml/min/1.73 m^2^ vs. 54.31 ± 27.64 ml/min/1.73 m^2^, p = 0.008). At admission, patients with better eGFR than 36.64 ml/min/1.73 m^2^ were discharged to their homes, mortality was higher with eGFR worse than 25.96 ml/min/1.73 m^2^, more RBC transfusion was needed if eGFR was lower than 49.61 ml/min/1.73 m^2^. Regulation of anticoagulation was examined extensively. Impaired kidney function at admission results higher in-hospital mortality in overt all-cause GIB and increases the need of RBC transfusion.

## Introduction

Kidneys are one of the most complex organs of the human body^1^. Glomerular filtration rate (GFR) is a widely used parameter for describing kidney function in clinical practice and research, meanwhile for everyday use estimated GFR (eGFR) is calculated using various formulas^2,3^.

With the help of eGFR, acute kidney injury (AKI) and chronic kidney disease (CKD) can be diagnosed and followed in a simple, fast, cost-efficient way. Based on the Global Burden of Disease Study, in 2017, there were 697.5 million cases of CKD worldwide. Almost 30% of these patients lived in China (132.3 million) and India (115.1 million), however the occurrence was lower in Europe than expected^4^. According to van Rijn et al*.*, in 2020, age-adjusted CKD prevalence was 5.5% in Spain and 5.6% in Iceland, Italy and UK, while 13.1% was in Estonia^5^. The pooled incidence of AKI ranged from 19.3% to 25.2%, based on which part of Europe was studied^6^.

The worldwide incidence of upper gastrointestinal bleeding (UGIB) is 40–150/100,000 people^7^. According to an evidence-based review by Kalman et al*.*, the CKD population is at an increased risk of UGIB and lower GIB (LGIB). They found the occurrence of various bleeding lesions, such as peptic ulcer, angioectasia, esophagogastric varices, diverticular diseases, and ischaemic colitis, more frequent among CKD patients^8^. UGIB patients with CKD or end-stage renal disease had three-fold higher all-cause mortality than individuals with normal renal function^9^. Additionally, dialysis, older age, diabetes mellitus (DM), history of ulcers, and cirrhosis were significantly associated with GIB in CKD^10^. In the meta-analysis of Hágendorn et al*.*, end-stage renal disease increased the mortality (OR 2.530, 95% CI 1.386–4.616, p = 0.002), the need for transfusion by 1.8 times, the rebleeding rate 2.5 more times, and length of hospitalisation (LOH) of GIB patients. They also emphasized the need for prospective studies^11^. According to a retrospective study by Cakmak et al*.*, AKI also lengthens hospital stay (4.1 ± 3.2 days vs. 6.7 ± 7.1 days/8.1 ± 7.5 days/9.4 ± 7.7 days depending on the severity of AKI), increases the need for intensive care unit (ICU) (0.2 ± 1.1 day vs. 2.5 ± 5.6 days), hospital costs, and overloads healthcare systems among UGIB patients^12^. In accordance with these, Grandholm et al*.* states that AKI itself results in an increased risk (relative effect 2.38, 95% CI 1.07–5.28) for clinically important GIB in adult ICU patients^13^. Although these studies highlight the association between loss of renal function and adverse outcomes in GIB, the classification ranges for CKD and AKI are not precise enough. There is a significant lack of knowledge regarding how eGFR influences specific clinical outcomes in GIB patients.

Pathophysiologically, chronic anemia, thrombocytopenia, deficiency of coagulation factors, uremic retention products, and uremia-induced platelet dysfunction may play a role in altered hemostasis among patients with renal disease^14^. In both acut and chronic uremia, platelets showed reduced adhesion between von Willebrand factor (vWF) and glycoprotein Ib. Acquired platelet storage pool defects, abnormal prostaglandin synthesis by platelets and endothelial cells, and increased nitric oxide levels affect platelet aggregation. A varied rheologic status due to anemia and the interference of uremic toxins on proteins such as fibrinogen or vWF has also been observed^15^.

Our study aimed to further evaluate the role of kidney function in several clinical outcomes of UGIB and LGIB patients.

## Methods

### Data source and study design

Our cohort analysis was based on 1160 patients from the Hungarian Gastrointestinal Bleeding Registry who were admitted to the First Department of Medicine, University of Pécs and the First Department of Internal Medicine, St. George University Teaching Hospital of County Fejér, Székesfehérvár, between 1st January 2019 and 6th April 2022. All adult (> 18 years) patients with overt signs of GIB (hematemesis, coffee-ground emesis, hematochezia, melena, blood in the nasogastric tube, and bleeding seen during endoscopy) from the two centres were included. Detailed data on patient characteristics, comorbidities, medication, treatments, procedures, and outcomes were pro- and retrospectively gathered in an online database (https://tm-centre.org/en/research/registries/gib-registry). The datasets generated and analysed during the current study is not publicly available in The Hungarian Gastrointestinal Bleeding Registry but are available from the corresponding author on reasonable request.

The manuscript was prepared accordingly Strengthening the Reporting of Observational Studies in Epidemiology (STROBE) 2007 Checklist (Table S1).

Ethical permission was granted by the National Public Health Center (24433-5/2019/EÜIG) in 2019. All methods were performed in accordance with the relevant guidelines and regulations, including the Declaration of Helsinki. All participants provided written, informed consent before being enrolled in the registry.

### Data quality

A multi-level data validation system was used to provide high-quality data. The system included four-level validation; all collected information was reviewed four times prior to the analysis. Data quality for all variables was 97.11% in the study population. For further details, see Supplementary Table S2.

### Representativeness of the cohort

In order to ensure that the analysed cohort’s distribution (n = 1160) does not differ from the original full cohort of 1215 patients, representability analysis was performed. No significant differences were found between the two groups. See Supplementary Fig. S1.

### Group formation for the descriptive analysis

The patients included were placed into groups based on the KDIGO 2024 Clinical Practice guideline according to their eGFR (ml/min/1.73 m^2^) values upon admission^16^. Five groups were created: patients with normal renal function (eGFR ≥ 90 ml/min/1.73 m^2^), mildly (90 ml/min/1.73 m^2^ > eGFR ≥ 60 ml/min/1.73 m^2^), moderately (60 ml/min/1.73 m^2^ > eGFR ≥ 30 ml/min/1.73 m^2^), severely (30 ml/min/1.73 m^2^ > eGFR ≥ 15 ml/min/1.73 m^2^) decreased renal function, and renal failure (eGFR < 15 ml/min/1.73 m^2^). Patient groups were also formed based on the source of GIB (regarding Treitz ligament) and used during descriptive analysis. Non-variceal upper (NVUGIB), variceal upper (VUGIB), small bowel (SGIB), LGIB, delayed iatrogenic, and intraprocedural iatrogenic GIB groups were differentiated.

### Definitions of laboratory parameters and outcomes

Overt signs of acute GIB included melena, hematemesis, or hematochezia detected by medical personnel, or iatrogenic bleeding observed during endoscopy. On admission, laboratory values included results gathered up to 36 h prior to or after the bleeding episode. Based on there, the CKD-EPI equation was used to estimate GFR values at admission. To evaluate patients’ baseline renal function, all of the available laboratory reports were overviewed from the year preceding the bleeding episode and we collected the most recent laboratory data unrelated to hospitalisation.

#### Primary outcomes

In-hospital mortality was quantified. Discharge home was established as leaving the hospital in agreement with the doctor after a proper procedure or course of in-hospital treatment. The need for endoscopic intervention was defined as the use of endoscopic hemostasis to control bleeding, including mechanical, chemical, or contact thermal therapy. In-hospital rebleeding was defined as newly-onset manifest GIB after cessation of melena, hematemesis, or hematochezia. LOH was defined as the number of days spent in the hospital due to GIB. Emergency surgery meant a need for any immediate surgical intervention. Any endoscopic examination during hospitalisation was counted. The need for ICU was the patient requiring admission to the ICU during hospitalisation.

#### Secondary outcomes

The detection of *Helicobacter pylori* (*H. pylori*) was based on serum antibody tests, stool antigen tests, rapid urease tests, urea breath tests, or histology results. Cancer as the source of bleeding was registered. The need for transfusion was defined as the use of at least one unit of the given concentrates, such as packed red blood cells (RBC), fresh frozen plasma or thrombocytes. The need for clotting factor concentrates was defined as administering a single or combination of factors intravenously at least once. Anticoagulation therapy, like cumarine, novel or direct oral anticoagulants (NOAC/DOAC) and low molecular weight heparin (LMWH) was registered.

### Statistical analysis

Descriptive statistical tools were used to summarize our data. In continuous variables, mean, standard deviation (SD), median, interquartile range (IQR) and minimum/maximum values were calculated, while in categorical variables, sample size and percentage were given. Welch’s t-test was used to examine the differences in the eGFR level between the groups determined by the outcomes. In this analysis, the Tukey’s Honest Significant Difference (TukeyHSD) test was used to compare the means across the levels of the factor variable after an ANOVA indicated a significant overall difference. The relationship between eGFR levels and continuous variables was analysed using Spearman’s rank correlation. Results were considered significant if p was less than 0.05. To determine whether a cut-off value can be specified for eGFR, which can predict the occurrence of a specific event, Receiver Operating Curve (ROC) analyses were applied. AUC classification levels were considered to be: excellent (0.9–1.0), good (0.8–0.9), fair (0.7–0.8), poor (0.6–0.7), and unsatisfactory (0.5–0.6). All analyses were performed in the R statistical environment (R version 4.1.2, R Core Team (2021). R: A Language and Environment for Statistical Computing. R Foundation for Statistical Computing, Vienna, Austria).

## Results

### Descriptive analysis

Altogether, 1160 GIB patients’ data were analysed from two centres, representing all GIB patients admitted into either of the two centres during the examination period. Due to the absence of reliable kidney function data, 55 patients were excluded. As for gender, 463 females and 697 males were involved. The mean age of the analysed cohort was 69.55 years (± 13.77), while it varied in a wide range depending on the kidney function at admission: normal: 55.24 years (± 11.84), mild: 67.42 years (± 13.24), moderate: 73.14 years (± 11.95), severe: 76.47 years (± 11.31), failure: 70.64 years (± 13.04). Statistically significant differences were found in the average age of the subjects’ when failure subgroup was compared to mild (p = 0.259) and moderate subgroups (p = 0.498). In case of any other comparison, no significant differences were found. Regarding the source of bleeding, in 619 cases (53%) NVUGIB was detected, 345 patients (30%) had LGIB, 104 individuals (9%) had VUGIB, and 24 (2.1%) had SGIB. In total, 53 (4.6%) delayed bleeding and 15 (1.3%) intraprocedural iatrogen bleeding cases were registered. Further patient characteristics (medical history, risk factors, comorbidities, laboratory parameters on admission, kidney status from the previous year, and medications at home) are detailed in Supplementary Table S3.

### Primary outcomes

#### In-hospital mortality

**Fig. 1:**
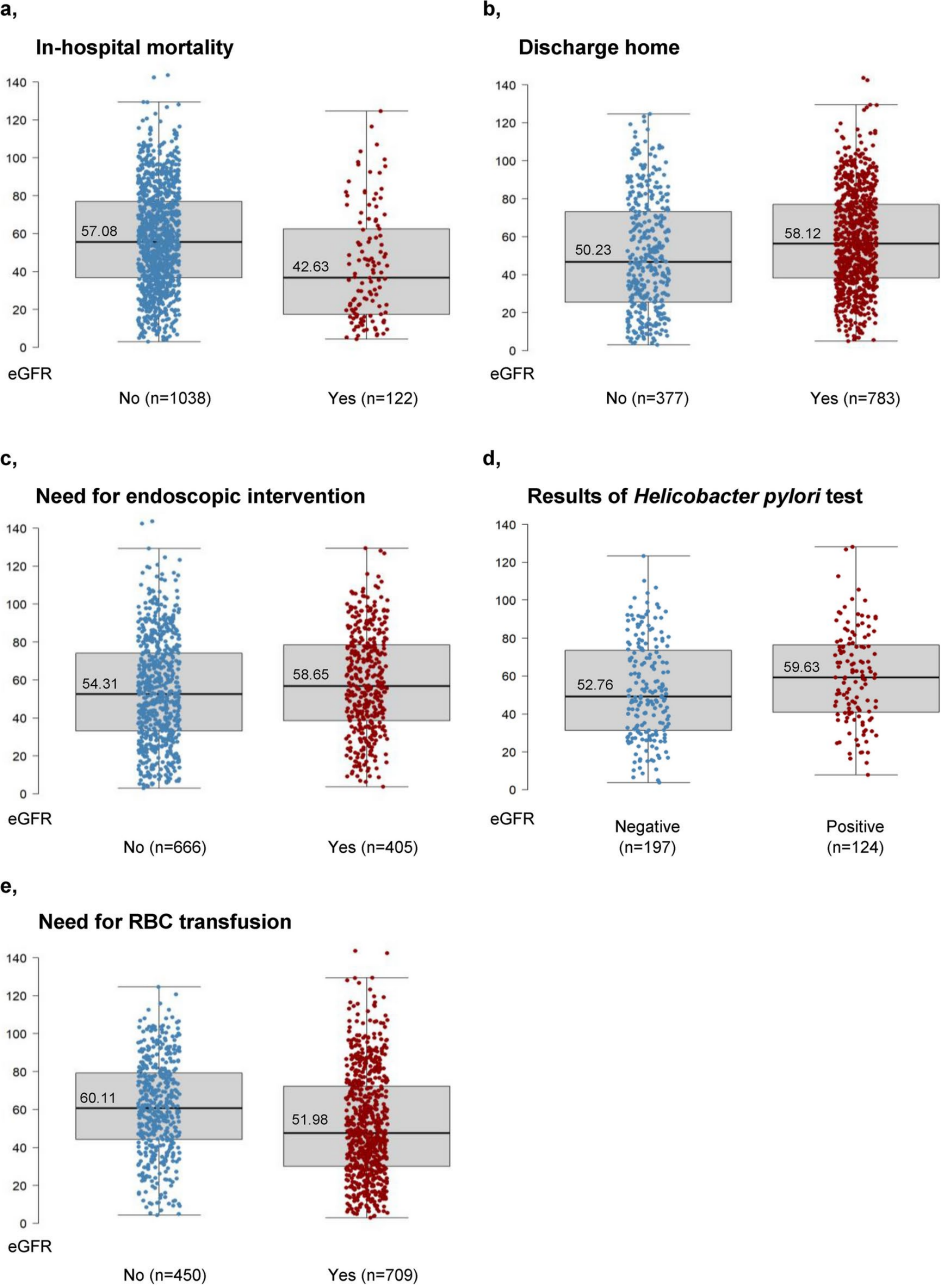
Kidney function distribution of patients for each outcome. In case of every figure, the scale represents the eGFR values (mL/min/1.73 m^2^) at admission, the thick line represents the median, the upper and lower side of the grey box represent Q1 and Q3, and the whiskers represents Q1 − 1.5 × IQR and Q3 + 1.5 × IQR. (**a**) In-hospital mortality. Significant difference was found in eGFR between individuals who compared to survival. (**b**) Discharge home. Patients with better kidney function was significantly more likely to be discharged home than others who died, transferred to other ward or left against medical advice. (**c**) Need for endoscopic intervention. 405 patient needed intervention (any endoscopic hemostatic method), and 666 not. The difference was significant. (**d**) Results of *Helicobacter pylori* test. Between the patients with positive and test, significant difference was found regarding kidney function at admission. (**e**) Need for RBC transfusion. 709 individuals needed RBC transfusion, while in 450 cases no anaemia was detected. The difference between the two groups was significant.

**Fig. 2:**
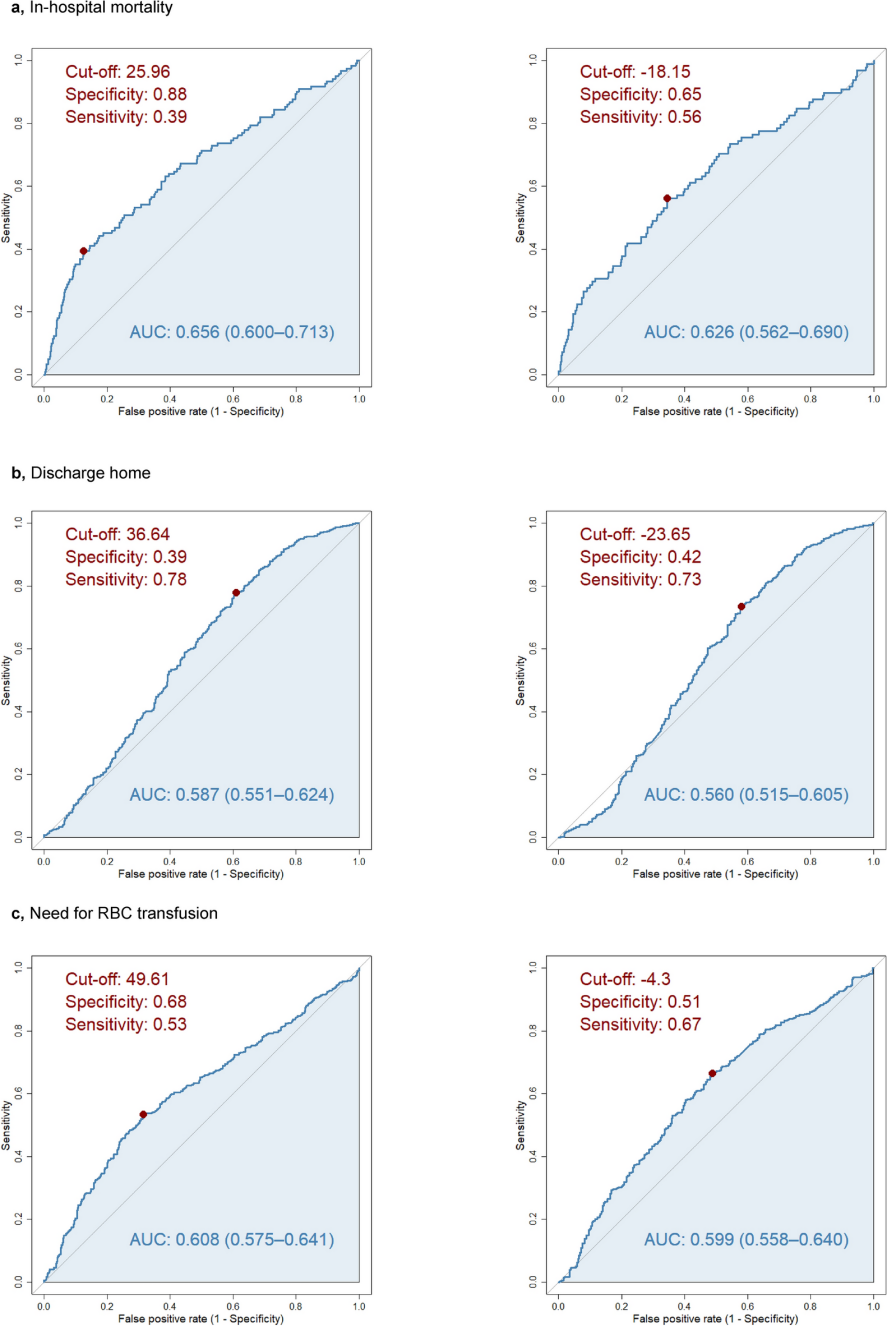
Cut-off values of study outcomes at admission based on the kidney function and change compared to the previous year. In both receiver operating characteristic (ROC) curves X-axis shows 1-specificity and Y-axis sensitivity, while cuf-off and area under curve (AUC) values are also noted. In every figure (**a–d**), which represents different study outcomes, the first plot shows the eGFR cuf-off value (ml/min/1.73 m^2^) at admission. The second plot represents the change in kidney function (expressed in percentage), when the laboratory parameter of kidney function at admission was compared to the same individual’s eGFR from the last year (365 + 1 days). Negative sign means decrease. (**a**) In-hospital mortality. If the patient’s eGFR at admission was less than 25.96 was more likely to die. When the same person’s actual kidney function decreased with 18.15% compared to its’ healthy status from the last year, the risk of lethal outcome was significantly higher (Fig. 3). (**b**) Discharge home. Above 36.64 eGFR value, the outcome was discharge home, rather than in-hospital mortality, transfer to other ward or leave against medical advice. The actual kidney function compared to a previous laboratory result from the previous year showed that 23.65% loss is associated with discharge home (Fig. 3). (**c**) Need for RBC transfusion. Patients with worse eGFR than 49.61 was more likely to receive RBC transfusion. 4.3% deterioration in kidney function at admission compared to the results from the previous increased the need for RBC transfusion (Fig. 3).

**Fig. 3:**
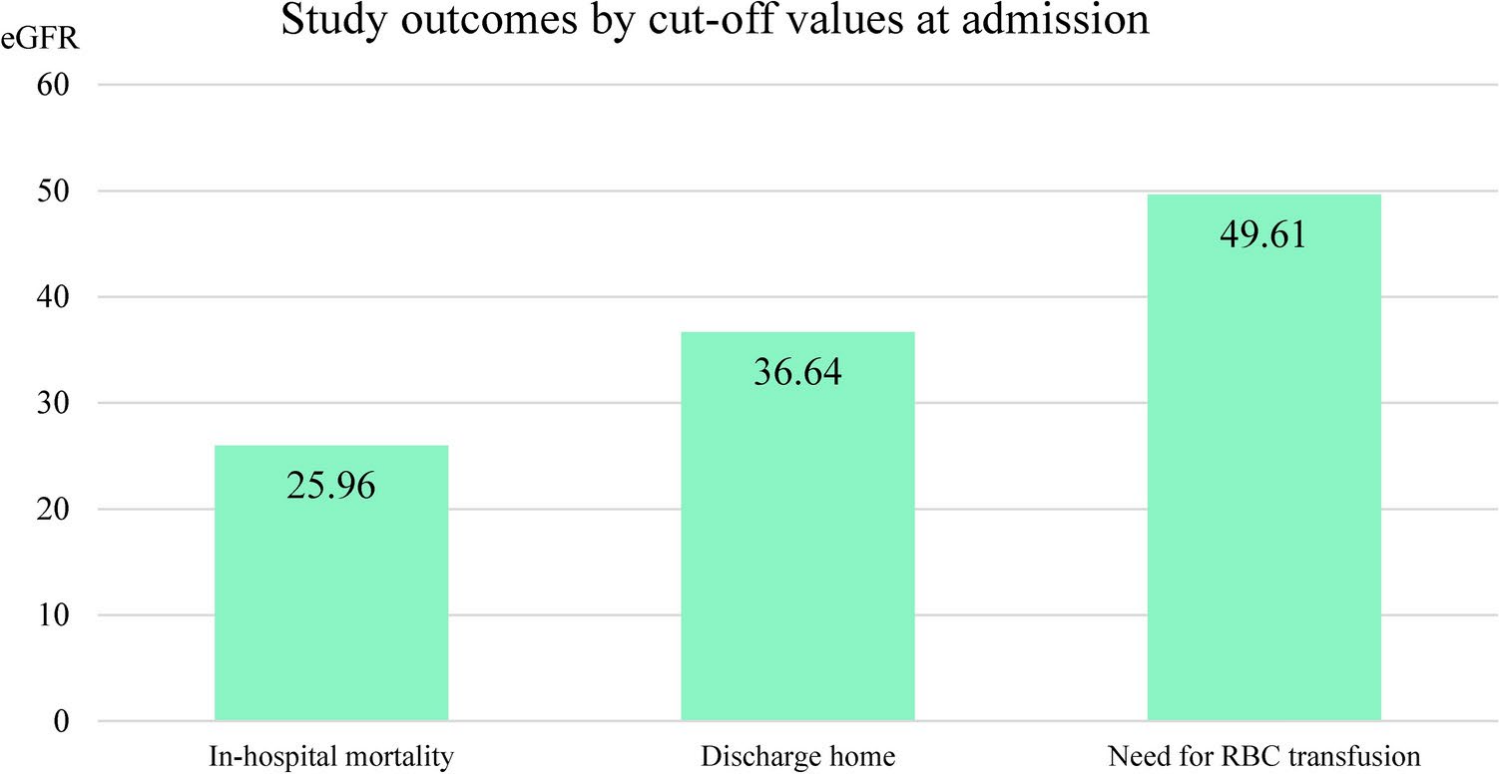
Summary of study outcomes by cut-off values at admission. In-hospital mortality. If the patient’s eGFR (ml/min/1.73 m^2^) at admission was less than 25.96 was more likely to die (Fig. 2a). Discharge home. Above 36.64 eGFR value, the outcome was discharge home, rather than in-hospital mortality, transfer to other ward or leave against medical advice (Fig. 2b). Need for RBC transfusion. Worse eGFR than 49.61 predicts RBC transfusion (Fig. 2c).

The in-hospital mortality was 11% (n = 122) in the analysed cohort (n = 1160). Significant difference (p < 0.001) was found in eGFR between individuals who died (n = 122, 11%, eGFR: 42.63 ± 28.78 ml/min/1.73 m^2^) and who survived (n = 1038, 89%, eGFR: 57.08 ± 26.62 ml/min/1.73 m^2^)) during hospitalisation due to GIB (Table 1, Fig. 1a). If the patient’s eGFR at admission was less than 25.96 ml/min/1.73 m^2^, the likelihood of death increased significantly (specificity: 0.88, sensitivity: 0.39, AUC: 0.656 (0.600–0.713)). When the same cohort’s actual kidney function (n = 796, mortality: n = 98, other outcome: n = 698) decreased by 18.15% compared to its prior status collected from the previous year’s (365 + 1 days) laboratory results, the risk of lethal outcome was significantly increased (specificity: 0.65, sensitivity: 0.56, AUC: 0.626 (0.562–0.690)) (Figs. 2a, 3).

**Table 1:** Outcomes of gastrointestinal bleeding patients according to kidney function on admission.

Outcome		Overall	Outcome missing	Outcome present	p value
In-hospital mortality	Number of cases	1160	1038	122	
	eGFR, mean (SD)	55.56 (27.21)	**57.08 (26.62)**	**42.63 (28.78)**	< 0.001
	eGFR, median (IQR)	53.64 (34.65, 75.77)	**55.58 (36.71, 77.00)**	**36.81 (17.52, 62.48)**	
	eGFR, min; max	3.06; 143.61	3.06; 143.61	4.31; 124.64	
Discharge home	Number of cases	1160	377	783	
	eGFR, mean (SD)	55.56 (27.21)	**50.23 (29.69)**	**58.12 (25.56)**	< 0.001
	eGFR, median (IQR)	53.64 (34.65, 75.77)	**46.76 (25.52, 73.25)**	**56.43 (38.29, 77.06)**	
	eGFR, min; max	3.06; 143.61	3.06; 124.64	5.01; 143.61	
Endoscopic intervention	Number of cases	1071	666	405	
	eGFR, mean (SD)	55.95 (27.33)	**54.31 (27.64)**	**58.65 (26.61)**	0.011
	eGFR, median (IQR)	53.85 (35.12, 76.60)	**52.52 (33.16, 74.13)**	**56.76 (38.60, 78.44)**	
	eGFR, min; max	3.06; 143.61	3.06; 143.61	3.79; 129.49	
*H. pylori* test positivity	Number of cases	321	197	124	
	eGFR, mean (SD)	55.41 (25.24)	**52.76 (25.44)**	**59.63 (24.45)**	0.017
	eGFR, median (IQR)	52.52 (35.40, 75.34)	**49.11 (31.35, 73.48)**	**59.16 (41.08, 76.01)**	
	eGFR, min; max	3.79; 128.13	3.79; 123.28	7.78; 128.13	
Need for RBC transfusion	Number of cases	1159	450	709	
	eGFR, mean (SD)	55.52 (27.19)	**61.11 (25.06)**	**51.98 (27.90)**	< 0.001
	eGFR, median (IQR)	53.63 (34.61, 75.71)	**60.72 (44.37, 79.13)**	**47.56 (30.07, 72.29)**	
	eGFR, min; max	3.06; 143.61	4.31; 124.64	3.06; 143.61	
	Number of cases	1155	1029	126	
Cumarine therapy	eGFR, mean (SD)	55.54 (27.22)	**56.91 (27.59)**	**44.37 (20.90)**	< 0.001
	eGFR, median (IQR)	53.63 (34.61, 75.71)	**55.54 (35.43, 77.78)**	**43.59 (30.31, 57.05)**	
	eGFR, min; max	3.06; 143.61	3.06; 143.61	6.73; 108.03	
	Number of cases	1155	1001	154	
NOAC/DOAC therapy	eGFR, mean (SD)	55.54 (27.22)	**57.57 (27.59)**	**42.37 (20.27)**	< 0.001
	eGFR, median (IQR)	53.63 (34.61, 75.71)	**56.36 (36.60, 78.34)**	**40.38 (25.41, 55.95)**	
	eGFR, min; max	3.06; 143.61	3.79; 143.61	3.06; 105.89	

*eGFR* estimated glomerular filtration rate (mL/min/1.73 m^2^), calculated based on the laboratory results on admission, *SD* standard deviation, *IQR* interquartile range, *Min* minimum, *Max* maximum, *H. pylori Helicobacter pylori*, *RBC* red blood cell, *NOAC/DOAC* novel or direct oral anticoagulation.

Significant values are in bold.

#### Discharge home

Patients with better kidney function (n = 783, eGFR: 58.12 ± 25.56 ml/min/1.73 m^2^) were significantly (p < 0.001) more likely to be discharged home than others (n = 377, eGFR: 50.23 ± 29.69 ml/min/1.73 m^2^) who died, transferred to another ward or left against medical advice (LAMA) (Table 1, Fig. 1b). Above 36.64 ml/min/1.73 m^2^ eGFR value, the outcome was found to be discharge to home rather than death, further hospitalisation on another ward or LAMA (specificity: 0.39, sensitivity: 0.78, AUC: 0.587 (0.551–0.624)). The actual kidney function compared to an earlier laboratory result from the previous year (n = 796, home: n = 524, other outcome: n = 272) showed that less than a 23.65% decrease in eGFR is associated with discharge home (specificity: 0.42, sensitivity: 0.73, AUC: 0.560 (0.515–0.605)) (Figs. 2b, 3).

#### Endoscopy/need for endoscopic intervention

No significant difference was found (p = 0.076) in kidney function when the group of patients who had endoscopy during hospitalisation (n = 1071, 92%, eGFR: 55.95 ± 27.33 ml/min/1.73 m^2^) was compared to those who did not (n = 89, 7.7%, eGFR: 50.89 ± 25.46 ml/min/1.73 m^2^).

During the procedure of patients having either gastroscopy or colonoscopy (n = 1071), in 405 cases (38% of patients, eGFR: 58.65 ± 26.61 ml/min/1.73 m^2^), an intervention was required, whereas in 666 cases (62%, eGFR: 54.31 ± 27.64 ml/min/1.73 m^2^) no endoscopic hemostatic method of any kind was used. Significant differences in eGFR values were found (p = 0.011) (Table 1, Fig. 1c).

#### In-hospital rebleeding

From 1160 patients, 1094 (94%) had no in-hospital rebleeding (eGFR 55.56 ± 37.21 ml/min/1.73 m^2^), while 66 patients (6%) bled (eGFR 55.66 ± 37.38 ml/min/1.73 m^2^). No significant difference was found in kidney function at admission between the two groups (p = 0.573).

#### Length of hospitalisation

There was no association between the kidney function registered at admission and the LOH (rho =  − 0.05, p = 0.072).

#### Emergency surgery

Thirty-nine patients (3.4%, eGFR: 49.39 ± 25.01 ml/min/1.73 m^2^) needed acute surgery. No significant difference was found in actual kidney function (p = 0.126) compared to those not requiring surgery (n = 1121, 97%, eGFR: 55.77 ± 27.27 ml/min/1.73 m^2^).

#### Need for ICU

One hundred and three patients (9%, eGFR: 52.09 ± 29.83 ml/min/1.73 m^2^) needed intensive care, and 1057 (91%, eGFR: 55.90 ± 26.93 ml/min/1.73 m^2^) were hospitalised in a high dependency unit or regular gastroenterology ward. There was no difference in eGFR values (p = 0.215).

### Secondary outcomes

#### *H. pylori* test and results

In case of 321 patients (28%) *H. pylori* test was performed, while 839 patients (72%) were not tested.

Whereas patients tested positive (n = 124, 39%, eGFR: 59.63 ± 25.24 ml/min/1.73 m^2^) had significantly higher eGFR values registered at hospital admission compared to infection-negative patients (n = 197, 61%, eGFR: 52.76 ± 25.44 ml/min/1.73 m^2^) (p = 0.017) (Table 1, Fig. 1d).

#### Need for RBC transfusion

RBC transfusion was needed in case of 709 individuals (61%, eGFR: 51.98 ± 27.90 ml/min/1.73 m^2^), while no RBC transfusion was required in 450 cases (39%, eGFR: 60.11 ± 25.06 ml/min/1.73 m^2^). The difference in kidney function of the two groups was significant (p < 0.001) (Table 1, Fig. 1e). Patients with worse eGFR than 49.61 ml/min/1.73 m^2^ were more likely to receive RBC transfusion (specificity: 0.68, sensitivity: 0.53, AUC: 0.608 (0.575–0.641)). Deterioration of kidney function by 4.3% at admission (specificity: 0.51, sensitivity: 0.67, AUC: 0.599 (0.558–0.640)) compared to previous year (n = 795, RBC transfusion: n = 502, no RBC transfusion: n = 293) increased the need for RBC transfusion (Figs. 2c, 3). Weak association was found between the kidney function and the amount of RBC transfusion (rho =  − 0.20, p < 0.001).

#### Regulation of blood coagulation

One thousand one hundred and fifty-five patents’ data was collected on cumarine, NOAC/DOAC or LMWH therapy. In case of cumarine, significant difference (p < 0.001) was found regarding eGFR at admission between individuals who took it (n = 126, 11%, eGFR: 44.37 ± 20.90 ml/min/1.73 m^2^) and those who did not (n = 1029, 89%, eGFR: 56.91 ± 27.59 ml/min/1.73 m^2^) (Table 1). Accordingly, cumarine takers were more likely to receive FFP (p = 0.046) or clotting factors (< 0.001) rather than patients who used other or no anticoagulants. eGFR was also significantly (p < 0.001) lower among NOAC/DOAC patients (n = 154, 13%, eGFR: 42.37 ± 20.27 ml/min/1.73 m^2^ vs. n = 1001, 87%, eGFR: 57.57 ± 27.59 ml/min/1.73 m^2^) (Table 1). In case of any anticoagulation (cumarine, NOAC/DOAC or LMWH), kidney function was found to be worse at admission (n = 370, 32%, eGFR: 46.17 ± 23.23 ml/min/1.73 m^2^ vs. n = 785, 68%, eGFR: 59.96 ± 27.85 ml/min/1.73 m^2^), and coagulation regulation therapy (FFP or clotting factors) needed to be carried out in more cases (< 0.001). Individuals needed clotting factors (p = 0.001), and clotting factors or FFP (p = 0.001) more often, when lower eGFR was registered at admission**.** There was no significant difference in eGFR values of patients receiving thrombocyte transfusion (n = 42, 4%, eGFR: 59.45 ± 27.18 ml/min/1.73 m^2^) vs. patients not receiving (n = 1118, 96%, eGFR: 55.41 ± 27.21 ml/min/1.73 m^2^) (p = 0.350).

#### Bleeding source identified as bleeding cancer

The source of bleeding was identified as cancer 106 times (9%). No significant difference was found in the kidney function of cancer-bleeding patients compared to those of patients bleeding due to other origins (eGFR: 59.803 ± 25.03 ml/min/1.73 m^2^ vs. 55.16 ± 27.41 ml/min/1.73 m^2^) (p = 0.074).

## Discussion

Our study aimed to further evaluate the role of kidney function on 8 primary and 5 secondary clinical outcomes in GIB patients. Significant differences were found in 3 primary outcomes: in-hospital mortality, discharge home, and need for endoscopic intervention; and in 3 secondary outcomes: *H. pylori* test positivity, need for RBC transfusion, and regulation of blood coagulation. Cut-off values of ROC curves presenting outcomes in eGFR and eGFR% change compared to previous year’s.

In our cohort, patients with reduced kidney function at admission were significantly more likely to die, used cumarine, NOAC/DOAC or any anticoagulation therapy at home in greater numbers, needed more RBC transfusion, clotting factors, and clotting factors or FFP. Better eGFR values were observed among individuals who were discharged home, had endoscopic hemostasis or whose *H. pylori* tests were positive. Our results are in agreement with Boyle and Johnston’s findings, which compared patients with moderate to severe CKD to healthy individuals and noted that acute UGIB was associated with an increased need for RBC transfusion and greater mortality^17^.

During literature research, no article was found in our topic, where renal function was expressed in eGFR, as a continuous variable. Mostly, CKD was characterized with GFR < 60 ml/min/1.73 m^2^ lasting longer than 3 months and AKI was defined as RIFLE stages R, I and F^11,12^. Due to this novelty, the numerical comparison of our results regarding in-hospital mortality to the previously published data is less accurate, however, the tendencies are consistent and clear. UGIB patients with CKD or end-stage renal disease had threefold higher risk to die than individuals with normal renal function^9^. A meta-analysis concluded the same regarding both UGIB and LGIB patients^11^. In-hospital mortality rate was 1.8 times higher among UGIB individuals with AKI^12^.

We found in accordance with published literature, better kidney function is associated with positive outcomes^11,12^. It is known that end-stage kidney disease causes bleeding diathesis, and this bleeding is independent of anticoagulation^18^. However, in our population this cannot be observed, according to the baseline characteristics patients, whose eGFR < 60 ml/min/1.73 m^2^ at admission, are on anticoagulant therapy in a greater portion. Our study did not prove that kidney deterioration would cause more severe GIB since there was no difference in rebleeding, need for ICU, or surgical intervention, which could all reflect the severity of bleeding. Furthermore, endoscopic interventions were fewer in patients with lower eGFR. The kidney function or function loss, compared to previous results found in GIB patients during the first assessment, seems predictive rather than in a causative relation with bleeding. The possibility, that kidney function deterioration leads to diffuse mucosal diathesis, cannot be proven by our analysis. The kidney function of patients with a malignant bleeding source, whose lesions were prone to bleed more diffusely than from a distinctive source, was similar to that of non-malignant bleeders.

Anticoagulation is an additive factor to those. The cumarine and NOAC/DOAC patients’ eGFR in our cohort were lower than without those anticoagulants. We also emphasize the predictor function of eGFR for GIB requiring more transfusion and elevating the risk of death. The higher need for clotting factor supplementation or FFP transfusion can be explained by higher blood loss and activated coagulation cascade found in kidney patients. According to Lutz et al., CKD may results either in thrombosis or in bleeding, depending on its severity. The worse the kidney function is, the more the procoagulatory state can be observed. Severe renal impairment (creatinine clearance < 20 ml/min or dialysis) causes prolonged bleeding time via platelet function default^19^ and accumulation of anticoagulants, all alternating bleeding^20^.

Interestingly, in our analysis, patients with significantly better kidney function were more likely to be presently infected by *H. pylori.* According to Pilotto et al*.*, advancing age is related to a higher frequency of chronic atrophic gastritis within less *H. pylori* positivity compared to younger age, where antralization of stomach mucosa has not taken place, resulting in less production of mucus for bacterial growth^21^. Kidney failure might result in other unknown-based unfavourable metabolic changes for bacteria, similar to mucosal atrophy found in the stomach more frequently at an elderly age. According to Shin et al*.*, the lower prevalence of *H. pylori* infection in CKD patients could also be explained by more frequent antacid and antibiotic intake among these individuals, elevated urea levels as inhibitors of the growth of *H. pylori* or increased inflammatory cytokines are associated with gastric mucosal damage, which makes *H. pylori* colonization difficult^22^. However a retrospective study found no association between the eradication of *H. pylori* and improvement in renal function^23^.

Using eGFR cut-off values at admission, a border can be drawn between the likely presence and the miss of each specific outcome in our cohort. In our study, these eGFR cut-off values selected patients likely to survive and to be discharged to their homes (> 36.64 ml/min/1.73 m^2^), patients not to survive (< 25.96 ml/min/1.73 m^2^), and patients who need RBC transfusion (< 49.61 ml/min/1.73 m^2^). In published literature no data regarding eGFR cut-off values can be found. However, due to low AUC values the strength of clinical prediction is limited.

In our study, kidney function at admission was compared to the same patient’s eGFR value registered during the last year. In terms of discharge home, 23.65% or less decrease in kidney function is associated with discharge home. This could be the rate of temporary AKI at the beginning of the GIB, which normalizes during proper hospitalisation. In general, 18.15% or more loss of kidney function increases the risk of lethality, calculated pioneeringly in this publication. This population was probably more fragile against GIB due to co-morbidities and having less reserve renal capacity. Based on these results, it is favourable to register earlier values in GIB patients.

Lastly, several frequent co-morbidities, as potential confounding factors primarily play a decisive role in kidney function, but based on literature data, they also affect GIB. Hypertension and CKD affect each other, while the former leads to deterioration in kidney function, progressive CKD can contribute to the worsening of high blood pressure^24^. In terms of GIB, portal hypertension is a life-threatening condition that leads to VUGIB^25^. Half of the type 2 and one third of type 1 DM patients develops symptoms of impaired renal function, especially elevated urinary albumin excretion^26^. In addition, DM is independently associated with VUGIB in Child–Pugh Class A cirrhotic patients^27^. Further studies should be performed, considering the co-morbidities.

### Strengths

Our analysis is innovative in presenting eGFR as continuous variable and provides a great range of GIB sources with countless outcomes. To our knowledge, based on literature research, patients’ kidney functions previously was classified into groups, like AKI, CKD, end-stage renal disease and healthy individuals.

According to the systematic review by Hágendorn et al*.*^11^, mortality, transfusion, rebleeding, and LOH were the most commonly discussed outcomes in GIB CKD population. It is also important to emphasize the presence of not commonly discussed outcomes, among our significant ones the need for endoscopic intervention, *H. pylori* test results, and the regulation of the coagulation system. Our analysis is innovative in defining specific eGFR cut-off values for several outcomes.

### Limitation

The main limitation of our study is the low AUC values of the eGFR cut-off values^28^. Between 0.5 and 0.6 the tests are unsatisfactory and 0.6–0.7 range represents poor results. Among the presented six ROC curves and AUC values, three are satisfactory. The exitus eGFR cut-off at admission and compared to the previous year, and the need for RBC transfusion eGFR cut-off at admission, whereas the AUC of the other three outcomes are less than 0.6, making interpretation questionable.

However, the cohort’s characteristics and recent hospitalisation were well registered, compared to the prevalence of GIB in Hungary (70–100/100.000 inhabitant/year)^29^, the case number is relatively small. Our cases were collected from only two tertiary centres, a university, and a teaching hospital. On the one hand, it is an advantage that the included patients received similar care in the hospitals. These are well-equipped institutes, and all necessary conditions are available to follow the most recent guidelines during the management of all GIB patients. Less equipped regions are missed, so the probability of negative outcomes during average care could be higher. 76% of our cohort was retrospectively collected, however the precise data collection made it possible to form six groups based on the source of bleeding (Table S3). No satisfactory analysis could be performed in order to compare these groups due to the small number of cases, especially among SBB, delayed, and intraprocedural iatrogenic patients. As detailed in the methods section, the definition of the laboratory parameters at admission made it possible to apply a ± 36 h time range from the onset of GIB. More precise laboratory measurements would be welcomed, but the availability and the costs are also considerable.

### Implication for general clinical practice

eGFR and GFR% change measured at the admission of GIB patients seem to carry information on the outcome of hospitalisation, however based on our results their clinical applicability may be limited by the moderate predictive accuracy. Kidney function along with other laboratory parameters, may point out fragile patients during management. Monitoring evaluating factors (e.g., fluid intake per os or intravenously, the amount of urine, arterial blood gas, pH) together with the laboratory parameters may help to notice alarming changes in time. In the case of individuals at higher risk, liberal transfusion and application of clotting factor supplementation might be considered earlier during management with longer monitoring.

These biomarkers’ relevance in case of individual patient management is not well-founded yet. At this point we are not able to answer weather the rate of eGFR reversal/improvement would have implication on patients’ outcomes. To answer this, more frequent and well-conceptualized blood sampling should be performed regarding parameters like creatinine, urea, and eGFR, preferably within clinical trials.

## Conclusion

Our study shows that impaired kidney function at admission leads to an increased rate of certain primary and secondary outcomes of GIB and it should be considered in all cases. Its’ main impact is on mortality. Patients with reduced eGFR values at admission are more likely to die, while the higher values are associated with discharge home. Lower laboratory results also predict the need for RBC transfusion and coagulation regulation by FFP transfusion or clotting factor supplementation. There might be an association between *H. pylori* positivity, the need for endoscopic intervention, and eGFR values, but further studies, preferably clinical trials, are needed.

## Supplementary Information

Supplementary Information.

## Data Availability

Detailed data on patient characteristics, comorbidities, medication, treatments, procedures, and outcomes were pro- and retrospectively gathered in an online database (https://tm-centre.org/en/research/registries/gib-registry). The datasets generated and analysed during the current study is not publicly available in The Hungarian Gastrointestinal Bleeding Registry but are available from the corresponding author on reasonable request.

## References

[1] Wallace, M. A. Anatomy and physiology of the kidney. *AORN J.* **68**(5), 800 (1998). 9829131 10.1016/s0001-2092(06)62377-6

[2] Levey, A. S., Titan, S. M., Powe, N. R., Coresh, J. & Inker, L. A. Kidney disease, race, and GFR estimation. *Clin. J. Am. Soc. Nephrol.* **15**(8), 1203–1212 (2020). 32393465 10.2215/CJN.12791019 PMC7409747

[3] National Kidney Foundation. *Estimated Glomerular Filtration Rate (eGFR)*. https://www.kidney.org/atoz/content/gfr#about-estimated-glomerular-rate-egfr (Accessed 13 July 2022).

[4] GBD Chronic Kidney Disease Collaboration. Global, regional, and national burden of chronic kidney disease, 1990–2017: A systematic analysis for the Global Burden of Disease Study 2017. *Lancet* **395**(10225), 709–733 (2020). 32061315 10.1016/S0140-6736(20)30045-3 PMC7049905

[5] van Rijn, M. H. C., de Pinho, N. A., Wetzels, J. F., van den Brand, J. A. J. G. & Stengel, B. Worldwide disparity in the relation between CKD prevalence and kidney failure risk. *Kidney Int. Rep.* **5**(12), 2284–2291 (2020). 33305122 10.1016/j.ekir.2020.09.040 PMC7710841

[6] Mehta, R. L. et al. International Society of Nephrology’s 0by25 initiative for acute kidney injury (zero preventable deaths by 2025): A human rights case for nephrology. *Lancet* **385**(9987), 2616–2643 (2015). 25777661 10.1016/S0140-6736(15)60126-X

[7] Gralnek, I. M. et al. Diagnosis and management of nonvariceal upper gastrointestinal hemorrhage: European Society of Gastrointestinal Endoscopy (ESGE) guideline. *Endoscopy* **47**(10), 1–46 (2015). 10.1055/s-0034-1393172 26417980

[8] Kalman, R. S. & Pedrosa, M. C. Evidence-based review of gastrointestinal bleeding in the chronic kidney disease patient. *Semin. Dial.* **28**(1), 68–74 (2015). 25215610 10.1111/sdi.12301

[9] Sood, P. et al. Chronic kidney disease and end-stage renal disease predict higher risk of mortality in patients with primary upper gastrointestinal bleeding. *Am. J. Nephrol.* **35**(3), 216–224 (2012). 22310659 10.1159/000336107 PMC7265418

[10] Lin, Y., Li, C., Waters, D. & Kwok, C. S. Gastrointestinal bleeding in chronic kidney disease patients: A systematic review and meta-analysis. *Renal Fail.* **45**(2), 2276908 (2023). 10.1080/0886022X.2023.2276908 PMC10796123 37955109

[11] Hágendorn, R. et al. Chronic kidney disease severely deteriorates the outcome of gastrointestinal bleeding: A meta-analysis. *World J. Gastroenterol.* **23**(47), 8415–8425 (2017). 29308001 10.3748/wjg.v23.i47.8415 PMC5743512

[12] Cakmak, U. et al. Effects of acute kidney injury on clinical outcomes in patients with upper gastrointestinal bleeding. *Renal Fail.* **38**(2), 176–184 (2016). 10.3109/0886022X.2015.1117923 26627631

[13] Granholm, A. et al. Predictors of gastrointestinal bleeding in adult ICU patients: A systematic review and meta-analysis. *Intens. Care Med.* **45**(10), 1347–1359 (2019). 10.1007/s00134-019-05751-6 31489445

[14] Eberst, M. E. & Berkowitz, L. R. Hemostasis in renal disease: Pathophysiology and management. *Am. J. Med.* **96**(2), 168–179 (1994). 8109602 10.1016/0002-9343(94)90138-4

[15] Fiaccadori, E. et al. Incidence, risk factors, and prognosis of gastrointestinal hemorrhage complicating acute renal failure. *Kidney Int.* **59**(4), 1510–1519 (2001). 11260415 10.1046/j.1523-1755.2001.0590041510.x

[16] Kidney Disease: Improving Global Outcomes (KDIGO) CKD Work Group. KDIGO: Clinical practice guideline for the evaluation and management of chronic kidney disease. *Kidney Int.* **2024**(105), S117–S314 (2024). 10.1016/j.kint.2023.10.018 38490803

[17] Boyle, J. M. & Johnston, B. Acute upper gastrointestinal hemorrhage in patients with chronic renal disease. *Am. J. Med.* **75**(3), 409–412 (1983). 6604455 10.1016/0002-9343(83)90341-8

[18] Zarka, F. et al. Risk of incident bleeding after acute kidney injury: A retrospective cohort study. *J. Crit. Care* **59**, 23–31 (2020). 32485439 10.1016/j.jcrc.2020.05.003

[19] Glorieux, G., Cohen, G., Jankowski, J. & Vanholder, R. Platelet/leukocyte activation, inflammation, and uremia. *Semin. Dial.* **22**(4), 423–427 (2009). 19708994 10.1111/j.1525-139X.2009.00593.x

[20] Lutz, J., Menke, J., Sollinger, D., Schinzel, H. & Thürmel, K. Haemostasis in chronic kidney disease. *Nephrol. Dial. Transplant.* **29**(1), 29–40 (2014). 24132242 10.1093/ndt/gft209

[21] Pilotto, A. & Franceschi, M. *Helicobacter pylori* infection in older people. *World J. Gastroenterol.* **20**(21), 6364–6373 (2014). 24914358 10.3748/wjg.v20.i21.6364 PMC4047322

[22] Shin, S. P., Bang, C. S., Lee, J. J. & Baik, G. H. *Helicobacter pylori* infection in patients with chronic kidney disease: A systematic review and meta-analysis. *Gut Liver* **13**(6), 628–641 (2019). 30970438 10.5009/gnl18517 PMC6860029

[23] Aljahdli, E., Almaghrabi, S. J., Alhejaili, T. L. & Alghamdi, W. Association between *Helicobacter pylori* eradication and kidney function in patients with chronic gastritis: A retrospective single-center study. *Cureus* **14**(1), e21621 (2022). 35228972 10.7759/cureus.21621 PMC8874340

[24] De Bhailis, Á. M. & Kalra, P. A. Hypertension and the kidneys. *Br. J. Hosp. Med. (Lond.)* **83**(5), 1–11 (2022). 35653320 10.12968/hmed.2021.0440

[25] Biecker, E. Portal hypertension and gastrointestinal bleeding: Diagnosis, prevention and management. *World J. Gastroenterol.* **19**(31), 5035–5050 (2013). 23964137 10.3748/wjg.v19.i31.5035 PMC3746375

[26] Akhtar, M., Taha, N. M., Nauman, A., Mujeeb, I. B. & Al-Nabet, A. D. M. H. Diabetic kidney disease: Past and present. *Adv. Anat. Pathol.* **27**(2), 87–97 (2020). 31876542 10.1097/PAP.0000000000000257

[27] Yang, C. H. et al. Diabetes mellitus is associated with gastroesophageal variceal bleeding in cirrhotic patients. *Kaohsiung J. Med. Sci.* **30**(10), 515–520 (2014). 25438683 10.1016/j.kjms.2014.06.002 PMC11916247

[28] Trifonova, O. P., Lokhov, P. G. & Archakov, A. I. Metabolic profiling of human blood. *Biomed. Khim.* **60**(3), 281–294 (2014). 25019391 10.18097/pbmc20146003281

[29] György, T. *Gatrointestinalis vérzés. 2018/2019.* https://docplayer.hu/111802305-Gastrointestinalis-verzes.html.

