# Supplementary material for: Renal dysfunction contributes to deteriorated survival outcomes in patients with upper and lower gastrointestinal bleeding: insights from a cohort study of 1160 cases
Source: Sci Rep. 2025 Jan 30;15:3781. doi: 10.1038/s41598-025-87969-7 (PMC11782606; doi:10.1038/s41598-025-87969-7)
Supplement: Supplementary file 1 — Supplementary Information. [file 41598_2025_87969_MOESM1_ESM.pdf]

Table S1. STROBE Statement - Checklist of items that should be included in reports of cohort studies.

	Item No	Recommendation	Page No.	Relevant text from manuscript
**Title and abstract**	1	(*a*) Indicate the study's design with a commonly used term in the title or the abstract	1	cohort analysis
		(*b*) Provide in the abstract an informative and balanced summary of what was done and what was found	2	Descriptive statistical tools were used to summarize our data. Among others, estimated glomerular filtration rate was used as continuous variable, mean, standard deviation, median, interquartile range and minimum/maximum values were calculated. Impaired kidney function at admission results higher in-hospital mortality in overt all-cause GIB and increases the need of RBC transfusion.
**Introduction**				
Background/rationale	2	Explain the scientific background and rationale for the investigation being reported	4	The worldwide incidence of upper gastrointestinal bleeding (GIB) is 40 - 150/100 000 people. According to an evidence-based review by Kalman *et al.*, the CKD population is at an increased risk of upper (UGIB) and lower GIB (LGIB). In the meta-analysis of Hágendorn *et al.*, end-stage renal disease increased the mortality, the need for transfusion, the rebleeding rate, and length of hospitalisation (LOH) of GIB patients. According to a retrospective study by Cakmak *et al.*, AKI also lengthens hospital stay, increases hospital costs, and overloads healthcare systems among upper GIB (UGIB) patients. In accordance with these, AKI itself results in an increased risk for GIB in adult intensive care unit (ICU) patients.
Objectives	3	State specific objectives, including any prespecified hypotheses	4	Our study aimed to further evaluate the role of kidney function in several clinical outcomes of UGIB and LGIB patients.
**Methods**				
Study design	4	Present key elements of study design early in the paper	4	Our cohort analysis was based on 1,160 patients from the Hungarian GIB Registry.
Setting	5	Describe the setting, locations, and relevant dates, including periods of	4	Our cohort analysis was based on 1,160 patients from the

		recruitment, exposure, follow-up, and data collection		Hungarian GIB Registry who were admitted to the First Department of Medicine, University of Pécs and the First Department of Internal Medicine, St. George University Teaching Hospital of County Fejér, Székesfehérvár, between 1st January 2019 and 6th April 2022.
Participants	6	(*a*) Give the eligibility criteria, and the sources and methods of selection of participants. Describe methods of follow-up	4	All adult (>18 years) patients with overt signs of GIB from the two centres were included.
		(*b*) For matched studies, give matching criteria and number of exposed and unexposed	N.A.	N.A.
Variables	7	Clearly define all outcomes, exposures, predictors, potential confounders, and effect modifiers. Give diagnostic criteria, if applicable	5	Definitions of laboratory parameters and outcomes
Data sources/ measurement	8*	For each variable of interest, give sources of data and details of methods of assessment (measurement). Describe comparability of assessment methods if there is more than one group	4	Detailed data on patient characteristics, comorbidities, medication, treatments, procedures, and outcomes were pro- and retrospectively gathered in an online database (https://tm-centre.org/en/research/registries/gib-registry).
Bias	9	Describe any efforts to address potential sources of bias	N.A.	N.A.
Study size	10	Explain how the study size was arrived at	4	Our cohort analysis was based on 1,160 patients from the Hungarian GIB Registry.
Quantitative variables	11	Explain how quantitative variables were handled in the analyses. If applicable, describe which groupings were chosen and why	5	The patients included were placed into groups according to their eGFR (mL/min/1.73m2) values upon admission. Five groups were created. Patient groups were also formed based on the source of GIB.
Statistical methods	12	(*a*) Describe all statistical methods, including those used to control for confounding	5-6	Descriptive statistical tools were used to summarize our data. In continuous variables, mean, standard deviation (SD), median, interquartile range (IQR) and minimum/maximum values were calculated, while in categorical variables, sample size and percentage were given.
		(*b*) Describe any methods used to examine subgroups and interactions	6	Welch's t-test was used to examine the differences in the eGFR level between the groups determined by the outcomes. The relationship between eGFR levels and continuous variables was analysed using Spearman's rank correlation. To determine

				whether a cut-off value can be specified for eGFR, which can predict the occurrence of a specific event, Receiver Operating Curve (ROC) analyses were applied.
		(*c*) Explain how missing data were addressed	N.A.	N.A.
		(*d*) If applicable, explain how loss to follow-up was addressed	N.A.	N.A.
		(*e*) Describe any sensitivity analyses	N.A.	N.A.
**Results**				
Participants	13*	(a) Report numbers of individuals at each stage of study—eg numbers potentially eligible, examined for eligibility, confirmed eligible, included in the study, completing follow-up, and analysed	6-11	Table 1, Online Resource 4
		(b) Give reasons for non-participation at each stage	6-11	Due to the absence of reliable kidney function data, 55 patients were excluded.<br>See each outcome detailed in the manuscript
		(c) Consider use of a flow diagram	N.A.	N.A.
Descriptive data	14*	(a) Give characteristics of study participants (eg demographic, clinical, social) and information on exposures and potential confounders	6-11	Table 1, Online Resource 4<br>See each outcome detailed in the manuscript
		(b) Indicate number of participants with missing data for each variable of interest	6-11	Table 1, Online Resource 4<br>See each outcome detailed in the manuscript
		(c) Summarise follow-up time (eg, average and total amount)	N.A.	N.A.
Outcome data	15*	Report numbers of outcome events or summary measures over time	6-11	Table 1, Online Resource 4
Main results	16	(*a*) Give unadjusted estimates and, if applicable, confounder-adjusted estimates and their precision (eg, 95% confidence interval). Make clear which confounders were adjusted for and why they were included	6-11	See each outcome detailed in the manuscript
		(*b*) Report category boundaries when continuous variables were categorized	5	See group formation for the descriptive analysis in the Methods section.
		(*c*) If relevant, consider translating estimates of relative risk into absolute risk for a meaningful time period	N.A.	N.A.
Other analyses	17	Report other analyses done—eg analyses of subgroups and interactions, and sensitivity analyses	6-11	Fig. 2 and Fig. 3.<br>See each outcome detailed in the manuscript
**Discussion**				
Key results	18	Summarise key results with reference to study objectives	12-14	Our study aimed to further evaluate the role of kidney function

				on 8 primary and 5 secondary clinical outcomes in GIB patients. Significant differences were found in 3 primary outcomes and in 3 secondary outcomes. Cut-off values of ROC curves presenting outcomes in eGFR and eGFR% change compared to previous year's.
Limitations	19	Discuss limitations of the study, taking into account sources of potential bias or imprecision. Discuss both direction and magnitude of any potential bias	13-14	The main limitation of our study is the low AUC values of the eGFR cut-off values. The case number is relatively small.
Interpretation	20	Give a cautious overall interpretation of results considering objectives, limitations, multiplicity of analyses, results from similar studies, and other relevant evidence	12-14	See detailed in interpretation.
Generalisability	21	Discuss the generalisability (external validity) of the study results	12-14	See detailed in interpretation.
**Other information**				
Funding	22	Give the source of funding and the role of the funders for the present study and, if applicable, for the original study on which the present article is based	3	Funding was provided by Tandem Funding of the University of Pécs (granted to Dr Hágendorn, KA-2021-10) and ÚNKP-22-3 New National Excellence Program of the Ministry for Innovation and Technology from the source of the National Research, Development and Innovation Fund (to BT - ÚNKP-23-3-II-PTE-1996).

Table S2. Data quality

	**EPIDEMIOLOGY**
1	Age
2	Gender
	Average uploaded data
	**RISK FACTORS**
3	Alcohol consumption
4	Smoking
	Average uploaded data
	**COMORBIDITIES**
5	Hypertension
6	Diabetes
7	Kidney function in the last year
	Average uploaded data
	**LABORATORY PARAMETERS ON ADMISSION**
8	Creatinine
9	eGFR
	Average uploaded data
	**OUTCOMES**
10	In-hospital rebleeding
11	Length of hospitalization
12	Outcome of admission - Home
13	Outcome of admission - Exitus
14	Outcome of admission - Going home on own responsibility
15	Outcome of admission - Admittes to other ward
16	Emergency surgery
17	Need for endoscopy
18	Need for endoscopic intervention
19	Need for ICU
20	H. pylori test
21	Bleeding source identified as cancer
22	Need for RBC transfusion
23	Need for FFP transfusion
24	Need for thrombocyte transfusion
25	Need for clotting factor
	Average uploaded data

TOTAL

OVERALL	UPLOADED DATA	%
1160	1160	100%
1160	1160	100%
2320	*2320*	*100%*

OVERALL	UPLOADED DATA	%
1160	938	81%
1160	916	79%
2320	*1854*	*80%*

OVERALL	UPLOADED DATA	%
1160	1159	100%
1160	1159	100%
1160	796	69%
3480	*3114*	*89%*

OVERALL	UPLOADED DATA	%
1160	1160	100%
1160	1160	100%
2320	*2320*	*100%*

OVERALL	UPLOADED DATA	%
1160	1160	100%
1160	1160	100%
1160	1160	100%
1160	1160	100%
1160	1160	100%
1160	1160	100%
1160	1160	100%
1160	1160	100%
1160	1160	100%
1160	1160	100%
1160	1160	100%
1160	1158	100%
1160	1159	100%
1160	1160	100%
1160	1160	100%
1160	1158	100%
18560	*18555*	*100%*

29000	***28163***	***97.11%***

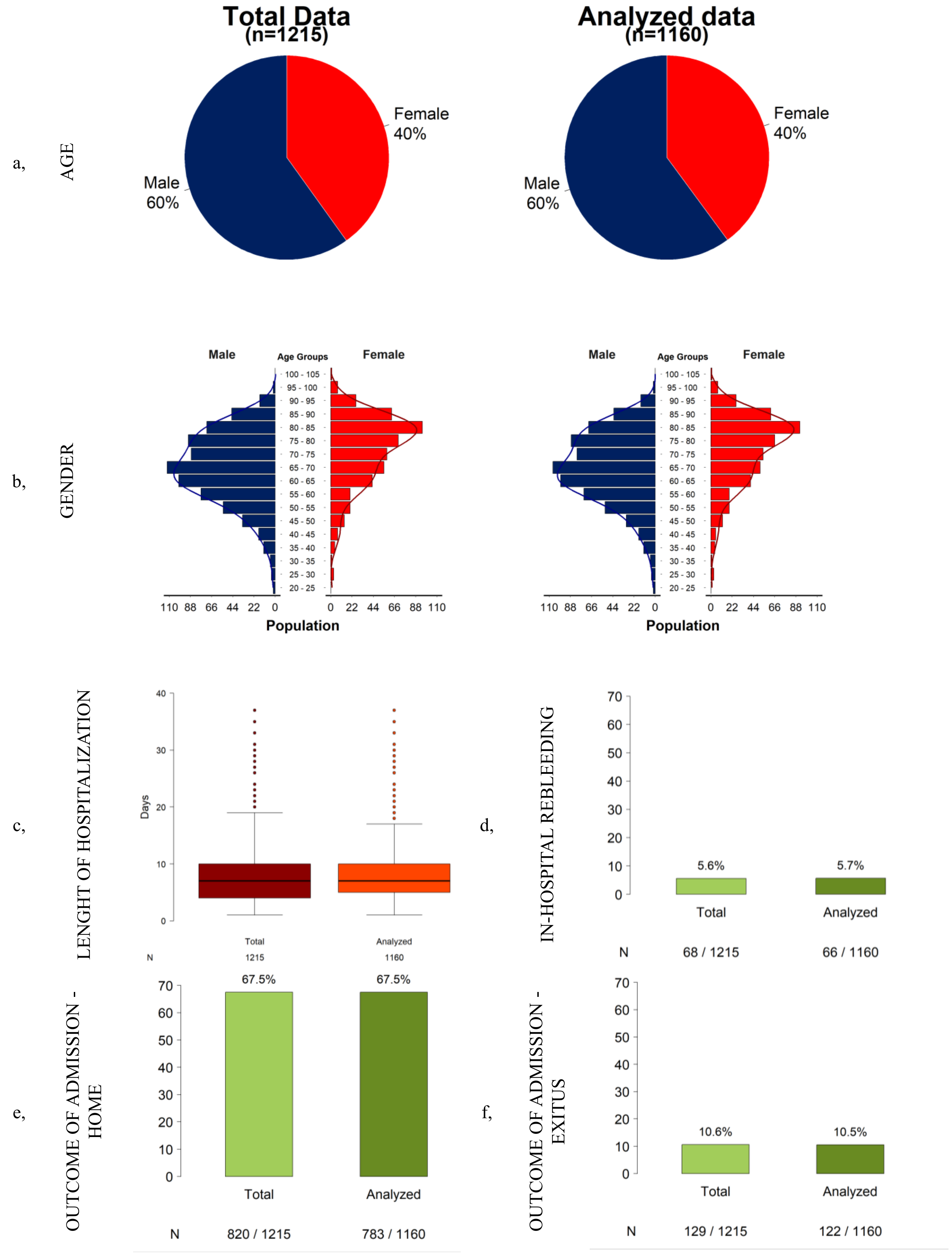
a,
AGE
Total Data
(n=1215)
Female
40%
Male
60%
Analyzed data
(n=1160)
Female
40%
Male
60%
b,
GENDER
Male
Age Groups
Female
100 - 105
95 - 100
90 - 95
85 - 90
80 - 85
75 - 80
70 - 75
65 - 70
60 - 65
55 - 60
50 - 55
45 - 50
40 - 45
35 - 40
30 - 35
25 - 30
20 - 25
110 88 66 44 22 0
0 22 44 66 88 110
Population
c,
LENGHT OF HOSPITALIZATION
Days
Total
Analyzed
N
1215
1160
d,
IN-HOSPITAL REBLEEDING
5.6%
5.7%
Total
Analyzed
N
68 / 1215
66 / 1160
e,
OUTCOME OF ADMISSION - HOME
67.5%
67.5%
Total
Analyzed
N
820 / 1215
783 / 1160
f,
OUTCOME OF ADMISSION - EXITUS
10.6%
10.5%
Total
Analyzed
N
129 / 1215
122 / 1160

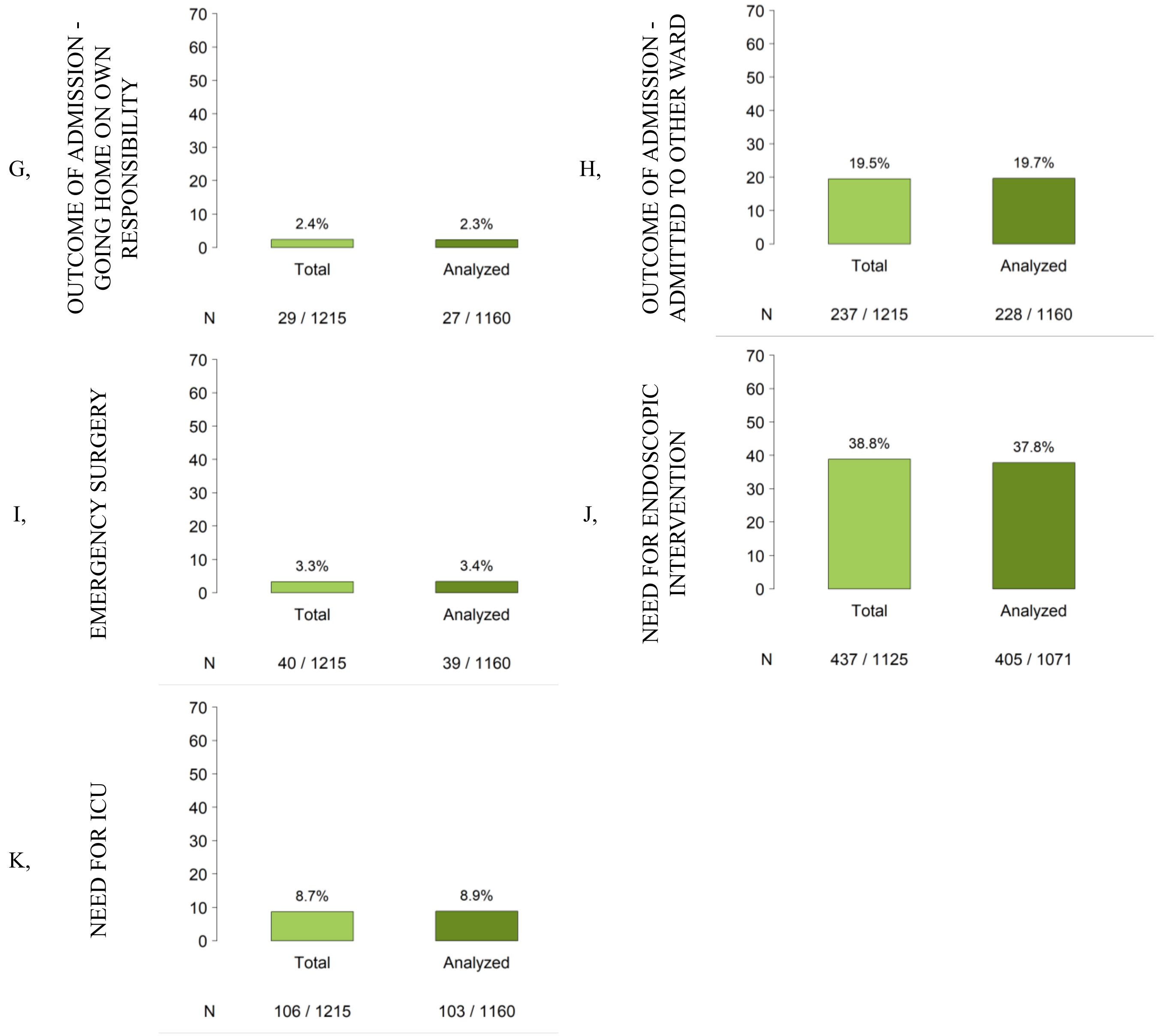

Figure S1 Representativity analysis

a, Pie chart of the age of the total data and the analyzed cohort

b, Age pyramide of the total data and the analyzed cohort. X-axis shows the populatin (n) and Y-axis represents the age groups in years.

c, Y-axis represents the number of days spent in the hospital. The first boxplot (brown) represents the total data, the second (orange) represents the analyzed cohort. The thick line represents the median, the upper and lower side of the boxes represent Q1 and Q3, and the whiskers represents the minimum (Q1-1,5xIQR) and maximum (Q3-1,5xIQR).

d, The Y-axis represents the number of patients who had symptoms of rebleeding during hospitalization. The first column (light green) represents the total data, the second (dark green) represents the analyzed data.

e, The Y-axis represents the number of patients who were discharged home after the hospitalization. The first column (light green) represents the total data, the second (dark green) represents the analyzed data.

f, The Y-axis represents the number of patients who died during hospitalization. The first column (light green) represents the total data, the second (dark green) represents the analyzed data.

g, The Y-axis represents the number of patients who interrupted the hospitalizaton and went home on their own responsibility. The first column (light green) represents the total data, the second (dark green) represents the analyzed data.

h, The Y-axis represents the number of patients who were admitted to another ward of the hospital. The first column (light green) represents the total data, the second (dark green) represents the analyzed data.

i, The Y-axis represents the number of patients who went throught emergency surgery during the hospitalization. The first column (light green) represents the total data, the second (dark green) represents the analyzed data.

j, The Y-axis represents the number of patients who needed endoscopic intervention during endoscopy. The first column (light green) represents the total data, the second (dark green) represents the analyzed data.
k, The Y-axis represents the number of patients who needed to be observed in ICU. The first column (light green) represents the total data, the second (dark green) represents the analyzed data.

Characteristic		Overall	Groups based on admission eGFR				
			Normal	Mild	Moderate	Severe	Failure
		1160 (100%)	144 (12%)	337 (29%)	449 (39%)	158 (14%)	72 (6%)
Hospital	Pécs	955 (82%)	121 (84%)	273 (81%)	367 (82%)	132 (84%)	62 (86%)
	Székesfehérvár	205 (18%)	23 (16%)	64 (19%)	82 (18%)	26 (16%)	10 (14%)
Data collection	Prospective	284 (24%)	46 (32%)	87 (26%)	106 (24%)	33 (21%)	12 (17%)
	Retrospective	876 (76%)	98 (68%)	250 (74%)	343 (76%)	125 (79%)	60 (83%)
Sex	Female	463 (40%)	50 (35%)	150 (45%)	161 (36%)	73 (46%)	29 (40%)
	Male	697 (60%)	94 (65%)	187 (55%)	288 (64%)	85 (54%)	43 (60%)
Age, mean (SD); years		69.55 (13.77)	55.24 (11.84)	67.42 (13.24)	73.14 (11.95)	76.47 (11.31)	70.64 (13.04)
Body weight, mean (SD), kg		77.18 (18.38)	75.12 (21.12)	76.62 (18.30)	77.62 (17.17)	77.38 (17.37)	80.97 (21.92)
Body height, mean (SD), cm		168.80 (9.29)	170.22 (9.25)	169.05 (9.57)	168.82 (9.12)	166.96 (9.50)	168.56 (8.27)
BMI, mean (SD); kg/m2		26.95 (5.53)	25.59 (5.93)	26.72 (5.33)	27.09 (5.09)	27.52 (6.23)	28.59 (6.23)
Admission	Inpatient	186 (16%)	26 (18%)	52 (15%)	58 (13%)	29 (18%)	21 (29%)
	Outpatient	974 (84%)	118 (82%)	285 (85%)	391 (87%)	129 (82%)	51 (71%)
Hemoglobin, mean (SD); g/L#		96.41 (30.67)	104.99 (28.46)	100.47 (29.78)	96.05 (31.43)	85.44 (28.41)	85.28 (30.05)
INR, mean (SD) #		1.79 (2.29)	1.56 (2.00)	1.47 (1.48)	2.03 (2.95)	1.78 (1.29)	2.29 (2.75)
CRP, mean (SD); mg/L#		30.29 (55.42)	26.77 (42.83)	23.05 (41.36)	26.45 (55.57)	45.63 (73.88)	65.38 (70.79)
WBC, mean (SD); G/L#		12.17 (7.49)	11.10 (4.47)	11.55 (6.51)	12.22 (8.36)	13.67 (8.85)	13.77 (7.15)
Neutrophils, mean (SD); %#		74.44 (10.81)	72.26 (11.91)	73.54 (10.13)	73.92 (10.84)	78.34 (10.06)	78.87 (10.18)
PLT, mean (SD); G/L#		272.87 (142.79)	241.32 (125.61)	282.59 (152.27)	282.80 (144.02)	262.32 (135.29)	250.38 (125.87)
Systolic BP, mean (SD); mmHg#		120.53 (27.67)	122.04 (25.77)	125.67 (26.44)	121.32 (28.09)	112.27 (28.67)	105.92 (23.41)
Diastolic BP, mean (SD); mmHg#		71.31 (15.46)	73.51 (13.37)	75.44 (15.26)	70.73 (15.10)	65.83 (15.25)	62.68 (16.12)
Pulse, mean (SD); 1/min#		94.05 (20.45)	99.63 (21.57)	95.74 (18.65)	93.18 (20.85)	90.85 (19.78)	87.44 (22.33)
Syncope on admission		81 (7.1%)	6 (4.2%)	31 (9.4%)	25 (5.7%)	10 (6.5%)	9 (13%)
Alcohol consumption		424 (45%)	72 (59%)	115 (42%)	158 (45%)	58 (44%)	21 (40%)
Smoking		220 (24%)	62 (52%)	66 (24%)	57 (17%)	22 (17%)	13 (25%)
Diabetes	Overall	332 (29%)	30 (21%)	98 (29%)	130 (29%)	48 (31%)	26 (36%)
	Type 1	3 (0.3%)	0 (0%)	1 (0.3%)	1 (0.2%)	0 (0%)	1 (1.4%)
	Type 2	327 (28%)	30 (21%)	97 (29%)	129 (29%)	47 (30%)	24 (33%)
	Type 3	2 (0.2%)	0 (0%)	0 (0%)	0 (0%)	1 (0.6%)	1 (1.4%)
Hypertension		808 (70%)	68 (47%)	200 (59%)	357 (80%)	122 (78%)	61 (85%)
Liver disease		246 (21%)	48 (33%)	69 (20%)	81 (18%)	28 (18%)	20 (28%)
Cirrhosis		165 (14%)	32 (22%)	48 (14%)	48 (11%)	22 (14%)	15 (21%)
Child Pugh stage	A	43 (3.7%)	8 (5.6%)	19 (5.6%)	11 (2.4%)	5 (3.2%)	0 (0%)
	B	63 (5.4%)	11 (7.6%)	15 (4.5%)	23 (5.1%)	9 (5.7%)	5 (6.9%)
	C	59 (5.1%)	13 (9.0%)	14 (4.2%)	14 (3.1%)	8 (5.1%)	10 (14%)
Ascites		109 (9.4%)	18 (12%)	31 (9.2%)	32 (7.1%)	18 (11%)	10 (14%)
Vascular disease	Overall	329 (28%)	29 (20%)	90 (27%)	135 (30%)	49 (31%)	26 (36%)
	Stroke	153 (13%)	15 (10%)	42 (12%)	59 (13%)	24 (15%)	13 (18%)
	TIA	39 (3.4%)	6 (4.2%)	12 (3.6%)	17 (3.8%)	2 (1.3%)	2 (2.8%)
	AMI	70 (6.0%)	9 (6.2%)	20 (5.9%)	28 (6.2%)	8 (5.1%)	5 (6.9%)
	Carotid AS	99 (8.5%)	7 (4.9%)	29 (8.6%)	39 (8.7%)	16 (10%)	8 (11%)
	Mesenteric AS	13 (1.1%)	1 (0.7%)	3 (0.9%)	8 (1.8%)	0 (0%)	1 (1.4%)
	Peripherial AS	63 (5.4%)	4 (2.8%)	18 (5.3%)	26 (5.8%)	11 (7.0%)	4 (5.6%)

Characteristic		Overall	Groups based on admission eGFR				
			**Normal**	**Mild**	**Moderate**	**Severe**	**Failure**
		1160 (100%)	144 (12%)	337 (29%)	449 (39%)	158 (14%)	72 (6%)
Thromboembolic disease	Overall	137 (12%)	8 (5.6%)	31 (9.2%)	69 (15%)	22 (14%)	7 (9.7%)
	DVT	74 (6.4%)	6 (4.2%)	15 (4.5%)	39 (8.7%)	10 (6.4%)	4 (5.6%)
	PVT	11 (0.9%)	0 (0%)	4 (1.2%)	3 (0.7%)	2 (1.3%)	2 (2.8%)
	PE	76 (6.6%)	5 (3.5%)	17 (5.0%)	38 (8.5%)	12 (7.6%)	4 (5.6%)
Ischemic heart disease		219 (24%)	14 (13%)	58 (22%)	89 (24%)	38 (32%)	20 (35%)
Heart valve disease		326 (29%)	16 (11%)	72 (22%)	141 (32%)	62 (40%)	35 (49%)
Cardiac valve replacement		27 (2.3%)	1 (0.7%)	4 (1.2%)	11 (2.4%)	5 (3.2%)	6 (8.3%)
Atrial fibrillation or flutter		268 (23%)	7 (4.9%)	52 (15%)	122 (27%)	60 (38%)	27 (38%)
Pacemaker implantation		52 (4.5%)	0 (0%)	9 (2.7%)	27 (6.0%)	12 (7.6%)	4 (5.6%)
Heart failure		116 (13%)	5 (4.7%)	19 (7.5%)	48 (14%)	30 (24%)	14 (26%)
Pulmonary disease		218 (19%)	22 (15%)	65 (19%)	83 (18%)	33 (21%)	15 (21%)
Peptic ulcer		137 (12%)	19 (13%)	43 (13%)	51 (11%)	15 (9.6%)	9 (12%)
Previous GIB		352 (30%)	43 (30%)	100 (30%)	141 (31%)	45 (29%)	23 (32%)
Malignant disease		138 (12%)	17 (12%)	41 (12%)	55 (12%)	19 (12%)	6 (8.5%)
H. pylori anamnesis	Positive	61 (5.4%)	9 (6.5%)	19 (5.7%)	22 (5.0%)	7 (4.7%)	4 (5.7%)
	Negative	98 (8.7%)	4 (2.9%)	26 (7.9%)	47 (11%)	13 (8.7%)	8 (11%)
	Not tested	967 (86%)	126 (91%)	286 (86%)	367 (84%)	130 (87%)	58 (83%)
Kidney failure last year*		660 (83%)	28 (29%)	194 (84%)	282 (93%)	105 (95%)	51 (89%)
CKD stage last year*	1	136 (17%)	67 (71%)	37 (16%)	20 (6.6%)	6 (5.4%)	6 (11%)
	2	306 (38%)	26 (27%)	161 (70%)	89 (29%)	20 (18%)	10 (18%)
	3	286 (36%)	2 (2.1%)	32 (14%)	185 (61%)	56 (50%)	11 (19%)
	4	50 (6.3%)	0 (0%)	1 (0.4%)	8 (2.6%)	28 (25%)	13 (23%)
	5	18 (2.3%)	0 (0%)	0 (0%)	0 (0%)	1 (0.9%)	17 (30%)
Hemodialysis last year*		18 (1.6%)	0 (0%)	0 (0%)	1 (0.2%)	4 (2.5%)	13 (18%)
On regular medication		1,037 (90%)	119 (83%)	290 (86%)	416 (93%)	146 (93%)	66 (92%)
Bleeding risk-increasing medication	Overall	718 (62%)	54 (38%)	196 (59%)	312 (70%)	110 (70%)	46 (64%)
	ASA	234 (20%)	17 (12%)	65 (19%)	107 (24%)	32 (20%)	13 (18%)
	Clopidogrel	140 (12%)	13 (9.0%)	41 (12%)	54 (12%)	23 (15%)	9 (12%)
	Coumarin	126 (11%)	3 (2.1%)	22 (6.6%)	71 (16%)	19 (12%)	11 (15%)
	DOAC	154 (13%)	2 (1.4%)	29 (8.7%)	71 (16%)	44 (28%)	8 (11%)
	Steroid	41 (3.5%)	5 (3.5%)	18 (5.4%)	11 (2.5%)	4 (2.5%)	3 (4.2%)
	LMWH	100 (8.7%)	12 (8.3%)	28 (8.4%)	37 (8.3%)	13 (8.3%)	10 (14%)
Gastroprotective medication	Overall	569 (49%)	74 (51%)	138 (41%)	220 (49%)	90 (57%)	47 (65%)
	PPI	444 (39%)	60 (42%)	113 (34%)	163 (36%)	71 (45%)	37 (51%)
	H2RA	141 (12%)	15 (10%)	29 (8.7%)	65 (15%)	22 (14%)	10 (14%)
Nephrotoxic medication	Overall	783 (68%)	82 (57%)	192 (57%)	317 (71%)	130 (83%)	62 (86%)
	Diuretics	537 (46%)	42 (29%)	116 (35%)	223 (50%)	108 (69%)	48 (67%)
	ACEI	447 (39%)	39 (27%)	109 (33%)	200 (45%)	67 (43%)	32 (44%)
	NSAID	165 (14%)	23 (16%)	53 (16%)	59 (13%)	21 (13%)	9 (12%)
	AB	41 (3.5%)	5 (3.5%)	10 (3.0%)	12 (2.7%)	8 (5.1%)	6 (8.3%)

Characteristic		Overall	Groups based on admission eGFR				
			Normal	Mild	Moderate	Severe	Failure
		1160 (100%)	144 (12%)	337 (29%)	449 (39%)	158 (14%)	72 (6%)
Bleeding source	NVUGIB	619 (53%)	67 (47%)	173 (51%)	230 (51%)	103 (65%)	46 (64%)
	VUGIB	104 (9.0%)	29 (20%)	35 (10%)	23 (5.1%)	11 (7.0%)	6 (8.3%)
	SBB	24 (2.1%)	3 (2.1%)	7 (2.1%)	10 (2.2%)	3 (1.9%)	1 (1.4%)
	LGIB	345 (30%)	34 (24%)	102 (30%)	153 (34%)	37 (23%)	19 (26%)
	delayed IA	53 (4.6%)	8 (5.6%)	16 (4.7%)	25 (5.6%)	4 (2.5%)	0 (0%)
	intraproc IA	15 (1.3%)	3 (2.1%)	4 (1.2%)	8 (1.8%)	0 (0%)	0 (0%)

Table S3. Characteristics of the cohort.

eGFR: estimated glomerular filtration rate. SD: standard deviation. BMI: body mass index. INR: international normalised ratio. CRP: C reactive protein. WBC: white blood cell count. PLT: platelet count. BP: blood pressure. TIA: transient ischaemic attack. AMI: acute myocardial infarction. AS: atherosclerosis. DVT: deep vein thrombosis. PVT: portal vein thrombosis. PE: pulmonary embolism. GIB: gastrointestinal bleeding. H. pylori: Helicobacter pylori. CKD: chronic kidney disease. ASA: aspirin. DOAC: direct oral anticoagulant. LMWH: low molecular weight heparin. PPI: proton pump inhibitor. H2RA: Histamin H2-receptor antagonist. ACEI: angiotensin-converting-enzyme inhibitor. NSAID: non-steroidal anti-inflammatory drug. AB: antibiotic. NVUGIB: non-variceal upper GIB. VUGIB: variceal upper GIB. LGIB: lower GIB. SBB: small bowel bleeding. IA: iatrogenic GIB. intraproc: intraprocedural. #: Admission values. *: Based on anamnesis and eGFR values obtained from the 365 days preceding the bleeding episode.
